# Efficacy and safety of upadacitinib maintenance therapy in patients with moderately to severely active Crohn’s disease: 2-year results from the U-ENDURE Long-Term Extension study

**DOI:** 10.1093/ecco-jcc/jjaf138

**Published:** 2025-07-24

**Authors:** Edward V Loftus, Jr, Geert D’Haens, Edouard Louis, Miguel Regueiro, Vipul Jairath, Fernando Magro, Hiroshi Nakase, Elena Dubcenco, Ana Paula Lacerda, Sharanya Ford, Tian Feng, Benjamin Duncan, Irina Fish, Colla Cunneen, Samuel I Anyanwu, Fernando Aponte, Jenny Griffith, Irina Blumenstein

**Affiliations:** Division of Gastroenterology and Hepatology, Mayo Clinic College of Medicine and Science, Rochester, MN, United States; Department of Gastroenterology and Hepatology, Amsterdam University Medical Centre, Amsterdam, The Netherlands; Department of Hepato-Gastroenterology and Digestive Oncology, University Hospital CHU of Liège, Liège, Belgium; Department of Gastroenterology and Hepatology, Cleveland Clinic Foundation, Cleveland, OH, United States; Department of Medicine, Western University, London, ON, Canada; Unit of Pharmacology and Therapeutics, Department of Biomedicine and Medicine, Porto, Portugal; Department of Gastroenterology, São João University Hospital Center, Porto, Portugal; CINTESIS@RISE, Department of Medicine, University of Porto, Porto, Portugal; Department of Gastroenterology and Hepatology, Sapporo Medical University School of Medicine, Sapporo, Japan; AbbVie, Inc, North Chicago, IL, United States; AbbVie, Inc, North Chicago, IL, United States; AbbVie, Inc, North Chicago, IL, United States; AbbVie, Inc, North Chicago, IL, United States; AbbVie, Inc, North Chicago, IL, United States; AbbVie, Inc, North Chicago, IL, United States; AbbVie, Inc, North Chicago, IL, United States; AbbVie, Inc, North Chicago, IL, United States; AbbVie, Inc, North Chicago, IL, United States; AbbVie, Inc, North Chicago, IL, United States; Department of Gastroenterology, Hepatology and Clinical Nutrition, Goethe University Frankfurt, University Hospital, Frankfurt, Germany

**Keywords:** Crohn’s disease, long-term extension, Janus kinase inhibitor, upadacitinib

## Abstract

**Background and Aims:**

The long-term efficacy and safety of upadacitinib in patients with moderate to severe Crohn’s disease (CD) were evaluated in the U-ENDURE Long-Term Extension (LTE) study. Here we report the results after 2 years of total maintenance treatment.

**Methods:**

U-ENDURE is an ongoing 240-week LTE study conducted at 243 sites across 43 countries (first patient enrolled in LTE 21 March 2019). Patients who completed the 52-week maintenance study continued their previously assigned treatment, once-daily upadacitinib 15 mg or upadacitinib 30 mg. Efficacy was analyzed at week 48 of the LTE; safety was analyzed in the cumulative study population (combined 52 week maintenance and 48 week LTE) and the LTE study population only (cutoff date: 19 December 2023).

**Results:**

From LTE week 0 to week 48, as-observed efficacy rates for clinical remission (per stool frequency/abdominal pain score, upadacitinib 15 mg: 78.3% to 82.9%; upadacitinib 30 mg: 84.7% to 76.6%; per CD Activity Index, upadacitinib 15 mg: 81.3% to 83.1%; upadacitinib 30 mg: 86.1% to 86.8%), endoscopic response (upadacitinib 15 mg: 59.6% to 67.1%; upadacitinib 30 mg: 71.2% to 69.6%), inflammatory biomarkers, and quality-of-life outcomes remained stable. The safety profile of the cumulative maintenance population observed through LTE week 48 was consistent with previous trials in the upadacitinib CD program. Treatment-emergent adverse event rates for the cumulative maintenance population were 283.1 and 273.4 events/100 patient-years for upadacitinib 15 mg and upadacitinib 30 mg, respectively. Event rates of serious treatment-­emergent adverse events were 16.0 events/100 patient-years for upadacitinib 15 mg and 14.6 events/100 patient-years for upadacitinib 30 mg. The most common adverse events of special interest (≥ 5.0 events/100 patient-years) were hepatic disorder, lymphopenia, creatine phosphokinase elevation, herpes zoster, and anemia. There was 1 treatment-emergent adverse event of suicide leading to death.

**Conclusion:**

Sustained clinical, endoscopic, quality-of-life, and biomarker outcomes were observed in patients who were initial responders to upadacitinib and completed 2 years of maintenance therapy, with no new safety signals identified.

**Clinical trial identifier:**

U-ENDURE; ClinicalTrials.gov number, NCT03345823.

## 1. Introduction

Crohn’s disease (CD) is a chronic, autoimmune, inflammatory bowel disease characterized by sporadic inflammation and ulceration throughout the gastrointestinal tract.^1^^,^^2^ Patients with CD experience diarrhea, abdominal pain, fatigue, and reduced quality of life (QoL), with uncontrolled intestinal inflammation leading to long-term complications such as fibrotic strictures and enteric fistulae.^2^ Though a number of conventional and advanced therapies are available for CD, including biologics and immunosuppressants, there remains an unmet need for safe and effective, long-term treatment options, as up to 40% of patients fail to respond to tumor necrosis factor (TNF) antagonists or lose response over time.^3^^,^^4^

Upadacitinib is an oral, reversible Janus kinase (JAK) inhibitor approved for treating moderately to severely active CD, ulcerative colitis, and many other indications.^5-7^ Previous studies demonstrated that upadacitinib induction (U-EXCEL/U-EXCEED) and maintenance therapy (U-ENDURE) achieved superior efficacy compared with placebo for the co-primary endpoints of clinical remission and endoscopic response at week 12 of induction and week 52 of maintenance.^8^ Upadacitinib-treated patients also attained higher levels of maintenance of clinical remission and corticosteroid-free clinical remission at week 52 of maintenance, compared with placebo-treated patients. No new safety signals were observed through week 52 of maintenance, consistent with the known safety profile of upadacitinib.^9^^,^^10^

U-ENDURE is a phase 3 long-term extension (LTE) study evaluating the long-term safety and efficacy of upadacitinib in patients with moderately to severely active CD who enrolled in the preceding induction and maintenance studies. This analysis reports safety and efficacy outcomes in patients who completed a minimum of 2 years of continuous upadacitinib treatment with the same dose during maintenance and LTE periods in the U-ENDURE studies.

## 2. Materials and methods

### 2.1 Study design and patients

**Figure 1.**
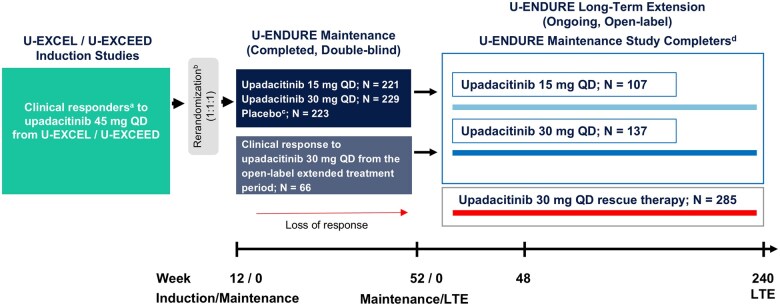
Key study design features of the U-ENDURE LTE study. APS, abdominal pain score; CD, Crohn’s disease; LTE, Long-Term Extension; QD, once-daily; SF, stool frequency. ^a^Patients who achieved a SF/APS clinical response (≥30% decrease in average daily very soft or liquid SF and/or in average daily APS and both not greater than baseline) to 12 weeks of upadacitinib 45 mg induction therapy were randomized to U-ENDURE maintenance. Patients who did not achieve a clinical response at induction week 12 could receive an additional 12 weeks of induction therapy during the induction extended treatment period (not shown). Clinical responders at week 24 were rerandomized into U-ENDURE maintenance. ^b^Rerandomization for U-ENDURE maintenance was stratified according to status with respect to previous failure of biologic therapy (yes or no), SF/APS clinical remission status (yes or no), and endoscopic response status (yes or no) at the end of the induction treatment. ^c^Patients from cohort 1 who received placebo treatment during the 52-week maintenance period continued to receive placebo during the LTE study but were not included in the analysis. Most of the placebo-treated patients who continued in the LTE from cohort 1 were rescued with upadacitinib 30 mg and/or CD-related medications; 29/51 were rescued with any rescue medications; 28/51 were rescued with open-label upadacitinib 30 mg; and 19/51-prematurely discontinued from the study for different reasons. ^d^Patients who completed the U-ENDURE 52-week maintenance were eligible for the subsequent LTE study and continued their previously assigned treatment of upadacitinib 15 mg or upadacitinib 30 mg. Efficacy was assessed from LTE week 0 to week 48.

The U-ENDURE (NCT03345823) study is an ongoing, 240-week, phase 3 LTE study at 243 centers across 43 countries involving patients with moderately to severely active CD (first patient enrolled 21 March 2019). Patient flow from previous studies of upadacitinib into U-ENDURE LTE is shown in Figure 1. The full details of the induction studies (U-EXCEL and U-EXCEED) and the maintenance study (U-ENDURE) have been previously published.^8^^,^^11^ Briefly, patients with moderately to severely active CD (defined as an average daily very soft or liquid stool frequency [SF] ≥ 4 and/or average daily abdominal pain score [APS] ≥ 2), and evidence of mucosal inflammation (defined as Simplified Endoscopic Score for CD [SES-CD] ≥ 6 [≥ 4 for patients with isolated ileal disease], excluding the presence of narrowing component), were eligible for inclusion in the induction studies. Patients who achieved a clinical response per SF/APS (≥ 30% decrease in average daily very soft or liquid SF and/or an average, daily APS and both not greater than baseline) after 12 weeks of upadacitinib 45 mg once-daily (QD) induction therapy were rerandomized to the 52-week maintenance study. Those who completed the U-ENDURE maintenance study were eligible for inclusion in the LTE study if they had an ileo-colonoscopy evaluation by a central reader and did not meet protocol-defined discontinuation criteria, which included adverse events (AEs) that occurred during the 52-week maintenance period necessitating study drug discontinuation due to malignancy, gastrointestinal (GI) perforation, high grade colonic dysplasia, recent cerebrovascular accidents, atypical laboratory values, and other criteria prespecified in the protocol.

At week 0 of LTE, all patients continued to receive their originally assigned double-blind treatment (placebo, upadacitinib 15 mg QD, upadacitinib 30 mg QD) until the end of the study. LTE treatment was unmasked after the last patient completed the 52 weeks of the maintenance study. Patients aged 65 years or older were recommended to decrease their upadacitinib dose from 30 mg to 15 mg. Patients with inadequate response or loss of response to their assigned treatment could be rescued with open-label upadacitinib 30 mg or protocol-specified CD-related medications. Interruptions of the study drug were required for the development of serious infection, herpes zoster, COVID-19, emergence of muscle-related symptoms suggestive of myositis or rhabdomyolysis, atypical laboratory values, elective and emergency surgeries, per criteria predefined in the protocol. The study drug was discontinued for the development of active tuberculosis, spontaneous GI perforation (other than due to appendicitis or mechanical injury), major adverse cardiovascular events (MACE, ie, acute myocardial infarction or stroke), malignancy, and thrombotic events.

Study visits occurred at LTE weeks 0, 4, and 12, and every 12 weeks thereafter until week 240. The endoscopic evaluation by SES-CD in the LTE study was performed every 48 weeks. All endoscopy videos were evaluated by central readers per the Endoscopy Image Review Charter. Seven independent readers, who were blinded to the site, patient data, the time point, and the date of the endoscopic procedure, were involved in endoscopy review and adjudication in case of disagreement between the local endoscopist and central reader.

The objective of the LTE study was to evaluate the long-term efficacy and safety of patients on continuous maintenance treatment with the same doses of upadacitinib.

### 2.2 Independent ethics committees and institutional review boards

The Independent Ethics Committee or Institutional Review Board at each study site approved the study protocol, informed consent forms, and recruitment materials before patient enrollment. The study was conducted in accordance with the International Conference for Harmonization guidelines, applicable regulations, and the Declaration of Helsinki. All patients provided written informed consent before screening.

### 2.3 Efficacy outcomes

Efficacy outcomes were evaluated through week 48 of the LTE study. Patients (*N* = 244) who completed the phase 3 upadacitinib U-ENDURE maintenance study were eligible to enter this LTE study for assessment of efficacy endpoints following treatment with upadacitinib (Figure S1). Of these individuals, 149 [61.1%] male patients and 95 [38.9%] female patients were treated with upadacitinib in the LTE study: 107 patients with upadacitinib 15 mg and 137 with upadacitinib 30 mg.

At week 48 of the LTE, the following endpoints were assessed: CD Activity Index (CDAI) clinical remission (CDAI < 150), SF/APS clinical remission (an average daily very soft or liquid SF ≤ 2.8, an average daily APS ≤ 1.0, and both values not greater than baseline of the induction study), maintenance of CDAI clinical remission (CDAI clinical remission at week 48 of the LTE study in patients who were in CDAI clinical remission at week 0 of the LTE study), maintenance of SF/APS clinical remission (SF/APS clinical remission at week 48 of the LTE study in patients who were in SF/APS clinical remission at week 0 of the LTE study), endoscopic response (decrease in SES-CD > 50% from baseline of the induction study [or for patients with an SES-CD of 4 at baseline of the induction study, at least a 2-point reduction from baseline]), endoscopic remission (SES-CD ≤ 4 and at least a 2-point reduction from baseline and no subscore > 1 in any individual variable), and maintenance of endoscopic remission (endoscopic remission at week 48 of the LTE study in patients who were in endoscopic remission at week 0 of the LTE study).

CDAI clinical remission, maintenance of CDAI clinical remission, endoscopic response, endoscopic remission, and maintenance of endoscopic remission were also assessed in a subgroup of patients who met the US FDA label criteria (ie, patients who had an inadequate response or intolerance to 1 or more TNF blockers, had a CDAI score ≥ 220 at induction baseline, and achieved clinical response-100 [CR-100] after 12 weeks of upadacitinib 45-mg treatment). Changes in inflammatory biomarkers, high-sensitivity C-reactive protein (hs-CRP), and fecal calprotectin (FCP) were evaluated from LTE week 0 through week 48 and expressed as the least squared (LS) mean change from the baseline (week 0) of induction.

Health-related quality-of-life (HRQoL) outcomes included the Inflammatory Bowel Disease Questionnaire (IBDQ), the Functional Assessment of Chronic Illness Therapy-Fatigue (FACIT-F), the 36-Item Short-Form (SF-36) Survey (version 2) Physical Component Score (PCS) and Mental Component Score (MCS), and the Work Productivity and Activity Impairment in CD (WPAI-CD) domains. IBDQ was quantified as IBDQ response (an increase in IBDQ score ≥ 16 points from baseline [week 0 of induction]), IBDQ remission (IBDQ score ≥ 170 points), and LS mean change from baseline of induction. FACIT-F, SF-36v2 PCS, SF-36v2 MCS, and WPAI-CD were quantified as LS mean change from baseline of induction.^12^

Occurrences of any CD-related hospitalizations and CD-related surgeries were reported by investigators during the trial and summarized by incidence rate calculated as the number of patients with the event divided by the time at risk in patient-years (PY) and are presented here as incidence of events per 100 PY (n/PYs [n/100 PY]). The surgical events occurring during the study were adjudicated by independent expert adjudicators blinded to treatment assignment to determine whether these events met prespecified definitions of CD-related events. CD-related hospitalizations were not adjudicated; the assessment was at the discretion of the investigator.

### 2.4 Administration of rescue therapy in patients who demonstrated an inadequate response

Patients who met the criteria for an inadequate response were eligible to receive rescue therapy (open-label upadacitinib 30 mg or protocol-specified CD-related medications, such as locally acting, oral, or intravenous corticosteroids, aminosalicylates, methotrexate, or CD-related antibiotics). Rescue treatment with CD-related medications was not withheld even if a patient did not meet the criteria for “inadequate response” if in the opinion of the investigator, failure to prescribe them could compromise the patient’s safety. Patients could also be discontinued at any time based on the investigator’s medical assessment. Patients with an inadequate response were required to demonstrate clinical symptoms including an average daily very soft or liquid SFS ≥ 4.0 and/or average daily APS ≥ 2.0, and an objective marker of inflammation (hs-CRP ≥ upper limit of normal and worse than the lowest value assessed during the maintenance and LTE studies, FCP ≥ 250 mg/kg and worse than the lowest value assessed in the present study, or SES-CD, excluding the narrowing component, ≥ 6 [≥ 4 for isolated ileal disease] as scored by the site investigator).

Once patients received rescue therapy, they were considered nonresponders for efficacy analyses of binary variables in the nonresponder imputation (NRI) analyses presented here; efficacy data collected after receiving rescue treatment were analyzed separately from the LTE study population. To evaluate the effect of open-label upadacitinib 30 mg rescue treatment, data from weeks 12 and 24 following its initiation were summarized, assessing endpoints among patients who received this treatment, including SF/APS clinical response (≥ 30% decrease in average daily SF and/or ≥ 30% decrease in average daily AP score and both not worse than baseline), enhanced clinical response (≥ 60% decrease in average daily SF and/or ≥ 35% decrease in average daily AP score, both not worse than baseline, or SF/APS clinical remission), SF/APS clinical remission, CDAI clinical remission, and absolute mean values of hs-CRP, and FCP.

### 2.5 Safety outcomes

Safety outcomes were assessed among 2 populations: the LTE study population only (*N* = 280) and among the cumulative maintenance population (*N* = 450; randomized upadacitinib responders). The LTE only population included patients randomized to upadacitinib 15 mg or upadacitinib 30 mg during the maintenance period and continued into the LTE; also included in this group were patients who had been exposed to other upadacitinib treatment algorithms prior to entering the LTE study (Figure S2). For the LTE only population, the AEs were assessed from LTE week 0 to week 48. The cumulative maintenance population included patients who entered the maintenance study and continued in the LTE on the same maintenance treatment dose of upadacitinib 15 mg or upadacitinib 30 mg; AEs were evaluated from maintenance week 0 to LTE week 48, with maximum exposure to upadacitinib at 285 weeks (Figure S2).

Safety outcomes included treatment-emergent adverse events (TEAEs), defined as any AEs that began or worsened in severity on or after the first dose of study drug in the maintenance treatment period and up to 30 days after the last dose. AEs of special interest were prespecified based on previous studies in patients receiving upadacitinib for CD and other indications or other JAK inhibitors^7–12^, and included serious infections, opportunistic infections, herpes zoster, malignancy excluding nonmelanoma skin cancer (NMSC), adjudicated MACE (defined as cardiovascular death, nonfatal myocardial infarction and nonfatal stroke), adjudicated venous thromboembolic events (VTEs, defined as deep vein thrombosis and pulmonary embolism [fatal and nonfatal]), adjudicated GI perforations, serious hypersensitivity reaction, and retinal detachment.

The cardiovascular and GI perforation events were adjudicated by an independent committee of physician experts in a blinded manner as defined by the Cardiovascular Adjudication Committee (CAC) or GI perforation adjudication charter. TEAEs were coded using the Medical Dictionary for Regulatory Activities (MedDRA), version 25.1. The severity of AEs was graded using the Common Terminology Criteria for Adverse Events, version 4.03.

### 2.6 Statistical analysis

For efficacy analyses, as-observed (AO) and NRI methods were used to summarize binary variables, while AO and modified AO (mAO) methods were used to summarize continuous variables. For NRI analysis on binary variables, patients were categorized as nonresponder after the initiation of any protocol rescue medications or missing data. AO analysis used all available data up to the initiation of open-label upadacitinib 30-mg rescue therapy and did not impute values for missing data. mAO analysis used all available data up to the initiation of any protocol-defined rescue medications and did not impute values for missing data. For binary variables, 95% confidence intervals (CI) for the response rate were based on the normal approximation to the binomial distribution. For continuous variables, 95% CI for the mean was based on the normal approximation. Statistical comparisons were not made between any treatment groups. As no statistical hypothesis testing was performed, multiplicity control was unnecessary.

Safety data were summarized descriptively, with no missing data imputed. TEAEs and AEs of special interest were summarized as exposure-adjusted event rates, (events/100 PY). Nonresponding patients were included in the safety analysis, except for patients who received rescue therapy. These patients were analyzed separately and are not presented here. All statistical analyses were completed using Statistical Analysis Software (version 9.4 or newer; SAS Institute, Cary, North Carolina, USA).

## 3. Results

### 3.1 Patient demographics and baseline characteristics

Of the 244 patients included in the LTE efficacy analysis, 107 and 137 patients continued their assigned doses of upadacitinib 15 mg and upadacitinib 30 mg when entering the LTE study. Among these patients, 70.1% (75/107) treated with upadacitinib 15 mg and 70.8% (97/137) treated with upadacitinib 30 mg completed LTE week 48 (Figure S3). There was no dose effect on the rates of discontinuation for patients who completed week 48 (upadacitinib 15 mg, 7.5%; upadacitinib 30 mg, 7.3%) and did not receive rescue therapy (upadacitinib 15 mg, 22.4%; upadacitinib 30 mg, 21.9%) (Figure S3). The most common reasons for discontinuation were other (nonspecified) reasons, TEAEs, and withdrawal of consent.

Baseline patient demographics and clinical characteristics of patients enrolled in the LTE study were generally well-balanced among the upadacitinib 15 mg or upadacitinib 30-mg-treated patients (Table S1).

**Table 1.** Disease characteristics at week 0 of U-ENDURE Long-Term Extension Study.

Characteristic, *n* (%) unless otherwise noted	Upadacitinib 15 mg *N* = 107	Upadacitinib 30 mg *N* = 137
**Medications at week 0 of LTE**		
*** Immunosuppressant use, yes***	5 (4.7)	1 (0.7)
*** Aminosalicylates use, yes***	29 (27.1)	30 (21.9)
*** Corticosteroid use, yes***	10 (9.3)	12 (8.8)
**CDAI, mean (SD)**	89.1 (67.1)	90.7 (61.0)
**SES-CD, mean (SD)**	6.1 (6.1) (*N* = 99)	4.7 (5.2) (N = 132)
**CDAI clinical remission, yes**	87 (81.3)	118 (86.1)
**SF/APS clinical remission, yes**	83 (78.3) (*N* = 106)	116 (84.7)
**Endoscopic response, yes**	59 (59.6) (*N* = 99)	94 (71.2) (*N* = 132)

APS, abdominal pain score; CDAI, Crohn’s disease activity index; SD, standard deviation; SES-CD, Simple Endoscopic Score for Crohn’s Disease; SF, stool frequency.

Data were calculated on nonmissing values. Week 0 of the LTE was equivalent to entry of the LTE study.

### 3.2 Efficacy

**Figure 2.**
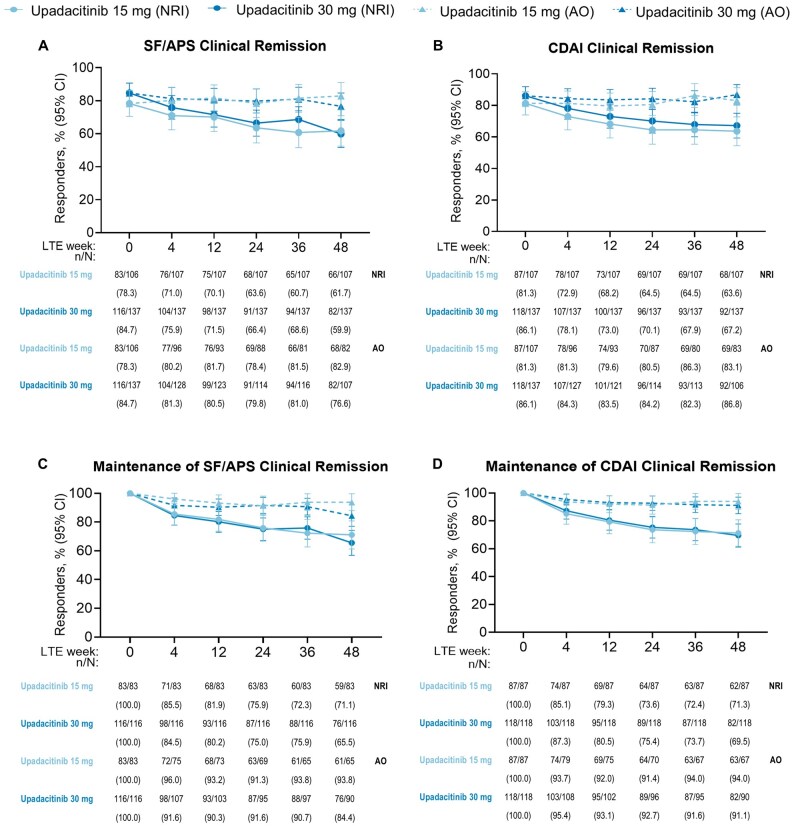
Clinical remission (A-B) and maintenance of clinical remission (C-D) in patients treated with upadacitinib through week 48 of the U-ENDURE LTE study. AO, as-observed; APS, abdominal pain score; CDAI, Crohn’s disease activity index; CI, confidence interval; LTE, long-term extension; NRI, nonresponder imputation; SF, stool frequency. AO analysis used all available data up to the initiation of open-label upadacitinib rescue and did not impute values for missing data. For NRI analysis on binary variables, patients were categorized as nonresponder after initiation of any protocol rescue medications or missing data. 95% CI for the response rate were based on the normal approximation to the binomial distribution.

**Figure 3.**
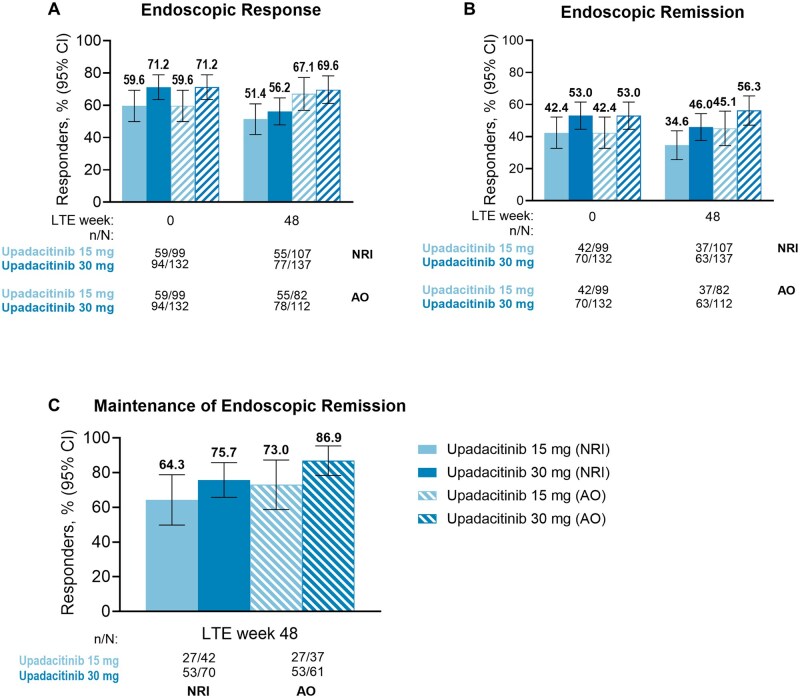
Endoscopic outcomes, including endoscopic response (A), endoscopic remission (B), and maintenance of endoscopic remission (C), in patients treated with upadacitinib through week 48 of the U-ENDURE LTE study. AO, as-observed; CI, confidence interval; LTE, long-term extension; NRI, nonresponder imputation. AO analysis used all available data up to initiating open-label upadacitinib rescue and did not impute values for missing data. For NRI analysis on binary variables, patients were categorized as nonresponder after initiating protocol rescue medications or missing data. 95% CI for the response rate were based on the normal approximation to the binomial distribution.

**Figure 4.**
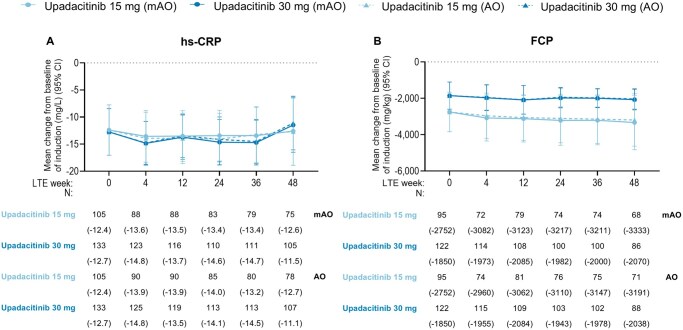
Inflammatory biomarkers, including hs-CRP (A) and FCP (B), in patients treated with upadacitinib through week 48 of the U-ENDURE Long-Term extension study. AO, as-observed; CI, confidence interval; FCP, fecal calprotectin; hs-CRP, highsensitivity C-reactive protein; LTE, long-term extension; mAO, modified as-observed. For mAO analysis on continuous variables, all available data up to the initiation of any protocol rescue medications were used, and values for missing data were not imputed. AO analysis used all available data up to the initiation of open-label upadacitinib rescue and did not impute values for missing data. 95% CI for the response rate were based on the normal approximation.

At week 0 of the LTE study, high rates of clinical and endoscopic outcomes were observed for patients on either upadacitinib 15 mg or upadacitinib 30 mg, with similar rates of patients for clinical remission (per SF/APS: 78.3%, 84.7%; per CDAI: 81.3%, 86.1%; Table 1). Patients treated with upadacitinib 30 mg (71.2%) tended to have numerically higher rates of endoscopic response compared with upadacitinib 15-mg-treated patients (59.6%) (Table 1). The high AO efficacy rates for SF/APS clinical remission (upadacitinib 15 mg: 78.3% to 82.9%; upadacitinib 30 mg: 84.7% to 76.6%, Figure 2A) and CDAI clinical remission (upadacitinib 15 mg: 81.3% to 83.1%; upadacitinib 30 mg: 86.1% to 86.8%, Figure 2B) observed at the beginning of the LTE period were sustained through the end of the 48-week period for both upadacitinib doses, with similar trends observed with more conservative NRI analysis. Most patients who were in SF/APS clinical remission at LTE week 0 maintained their status through week 48 (Figure 2C), with similar results observed for maintenance of CDAI clinical remission (Figure 2D). Rates of endoscopic response (upadacitinib 15 mg, AO: 59.6% to 67.1%, upadacitinib 30 mg: 71.2% to 69.6%) and endoscopic remission (upadacitinib 15 mg, AO: 42.4% to 45.1%, upadacitinib 30 mg: 53.0% to 56.3%) remained consistent from LTE week 0 compared with week 48 when evaluated by either NRI or AO analyses (Figure 3A-B). Most patients in endoscopic remission at LTE week 0 were able to sustain their response through week 48 (Figure 3C). Clinical and endoscopic efficacy results among the subpopulation of TNF-IR patients who met the US label criteria of moderately to severely active CD were consistent with the overall population (Figure S4). Mean changes in hs-CRP from baseline of induction remained stable through LTE week 48 (Figure 4A). Similar results were observed for FCP, with stable mean changes from baseline of induction through LTE week 48 (Figure 4B).

**Figure 5.**
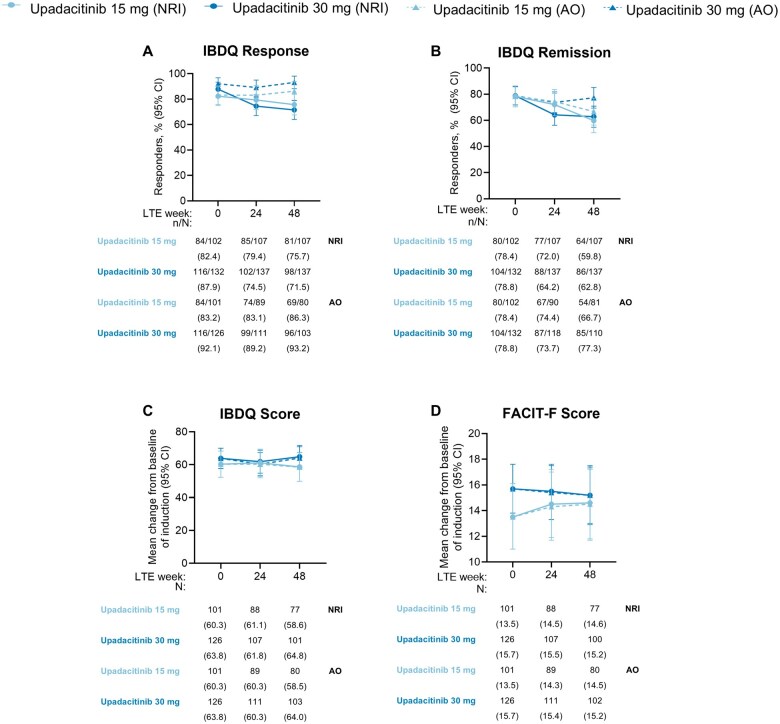
IBDQ and FACIT-F scores in patients treated with upadacitinib through week 48 of the U-ENDURE Long-Term extension study. AO, as-observed; CI, confidence interval; FACIT-F, functional assessment of chronic illness Therapy-Fatigue; IBDQ, inflammatory bowel disease questionnaire; LTE, long-term extension; NRI; nonresponder imputation. Data were presented as the percentage of patients who achieved (A) IBDQ response, (B) IBDQ remission, (C) mean change from baseline of induction in IBDQ score, or (D) mean change from baseline of induction in FACIT-Fatigue score. AO analysis used all available data up to the initiation of open-label upadacitinib rescue therapy and did not impute values for missing data. For NRI analysis on binary variables, patients were categorized as nonresponders after initiation of any protocol rescue medications or missing data. 95% CI was calculated based normal approximation.

The rates of IBDQ response and IBDQ remission generally remained stable from LTE week 0 through week 48 (Figure 5A-B). IBDQ scores remained consistent, with mean changes from baseline of induction remaining stable through LTE week 48 (Figure 5C). Improvements in fatigue were also sustained through LTE week 48 (Figure 5D). Improvements in SF-36v2 PCS and MCS also remained stable among upadacitinib-treated patients from LTE week 0 through week 48 (­Figure S5A-B). Similar results were observed with work productivity and performance of daily activities in patients treated with upadacitinib throughout the LTE study, assessed by a consistency in the mean change from baseline of induction in all 4 WPAI-CD domains, including overall work impairment, activity impairment, absenteeism, and presenteeism (Figure S5C-F). Results for NRI efficacy were reported in Figures 2-5 and Figure S5.

Incidence rates for CD-related hospitalizations and CD-­related surgeries were assessed among upadacitinib-treated patients during the 48-week LTE study, with rates of 1.5, 1.5/100 PY for upadacitinib 15 mg- and 3.1, 1.2/100 PY for upadacitinib 30-mg-treated patients, respectively (Table S2).

To evaluate the effect of open-label upadacitinib 30-mg rescue treatment, data from weeks 12 and weeks 24 after initiation of open-label upadacitinib 30 mg rescue therapy in patients (*N* = 110 and *N* = 175 originally allocated to the upadacitinib 15 mg and placebo groups, respectively) during the LTE period were summarized. Increased rates for SF/APS clinical response (placebo, 89.7%; upadacitinib 15 mg, 90.0%), enhanced clinical response (placebo, 82.8%; upadacitinib 15 mg, 86.7%), clinical remission (per SF/APS: placebo, 60.0%; upadacitinib 15 mg, 55.6%; per CDAI: placebo, 56.3%; upadacitinib 15 mg, 59.1%) were observed at 24 weeks after initiation of rescue treatment, in addition to lower absolute means of hs-CRP (placebo, 9.0 mg/L; upadacitinib 15 mg, 9.9 mg/L) and FCP (placebo, 1173 mg/kg; upadacitinib 15 mg, 909 mg/kg) (Figure S6).

### 3.3 Safety

In the long-term safety analysis population only, we assessed 280 patients treated with upadacitinib during the LTE study, representing 197.0 PY for upadacitinib 15 mg and 321.6 PY for upadacitinib 30 mg (Table 2). The median exposure for upadacitinib 15 mg was 94.3 weeks (min, max = 1.0, 229.6 weeks) and 91.3 weeks (min, max = 0.6, 227.3 weeks) for upadacitinib 30 mg (Table 2). In the cumulative safety analysis population, we assessed 450 patients treated with upadacitinib during the maintenance or LTE studies, representing 350.7 PY for upadacitinib 15 mg and 432.8 PY for upadacitinib 30 mg. The median exposure for upadacitinib 15 mg was 51.6 weeks (min, max = 1.3, 285.3) and 103.1 weeks (min, max = 1.1, 279.9 weeks) for upadacitinib 30 mg (Table 3).

**Table 2.** Treatment-emergent adverse events in patients treated with upadacitinib from week 0 to week 48 of the U-ENDURE Long-Term extension study.

Adverse event, E (E/100 PYs)^a^	**LTE population^b^**	
	Upadacitinib 15 mg *N* = 107; PYs = 197.0 Median exposure = 94.3 weeks Min, max= 1.0, 229.6	Upadacitinib 30 mg *N* = 173; PYs = 321.6 Median exposure = 91.3 weeks Min, max= 0.6, 227.3
Overview of TEAEs		
*Any AE*	447 (226.9)	775 (241.0)
*Any AE leading to the discontinuation of the study drug*	6 (3.0)	13 (4.0)
*Any AE with a reasonable possibility of being related to the study drug^c^*	112 (56.9)	199 (61.9)
*COVID-19 infection*	21 (10.7)	61 (19.0)
*Severe AE*	19 (9.6)	29 (9.0)
*Serious AE*	19 (9.6)	33 (10.3)
*Deaths*	1 (0.5)^d^	0
AEs of Special Interest		
*Serious infection*	3 (1.5)	8 (2.5)
*Opportunistic infection, excluding tuberculosis and herpes zoster^e^*	1 (0.5)	1 (0.3)
*Active tuberculosis*	0	0
*Herpes zoster*	3 (1.5)	15 (4.7)
*Adjudicated gastrointestinal perforation*	3 (1.5)^f^	0
*Anemia*	4 (2.0)	15 (4.7)
*Neutropenia*	9 (4.6)	6 (1.9)
*Lymphopenia*	12 (6.1)	29 (9.0)
*Creatine phosphokinase elevation*	16 (8.1)	16 (5.0)
*Hepatic disorder*	13 (6.6)	29 (9.0)
*Renal dysfunction*	0	2 (0.6)
*Malignancies excluding NMSC^g^*	2 (1.0)	1 (0.3)
*Any NMSC*	0	3 (0.9)
*Lymphoma*	0	0
*Adjudicated MACE*	0	0
*Adjudicated VTE*	0	1 (0.3)^h^
*Serious hypersensitivity reactions*	0	0
*Retinal detachments*	0	0

AE, adverse event; E, event; MACE, major adverse cardiovascular event; NMSC, nonmelanoma skin cancer; PY, patient-year; TEAE, treatment-emergent adverse event; VTE, venous thromboembolic event.

a Data was expressed as exposure-adjusted E/100 PY = number of events per 100 PY. TEAEs were defined as those that occurred on or after the first dose of study drug administration and within 30 days after the last dose of study drug administration per defined cohort/treatment group. Data cutoff date: 19 December 2023.

b Patients in the long-term extension population received up to 229 weeks (2 years) of upadacitinib maintenance therapy. This population included patients only in the LTE study, from LTE week 0 to week 48.

c As assessed by the study investigator.

d One event of suicide with no reasonable possibility of being related to the study drug as assessed by the investigator.

e Opportunistic infections, excluding tuberculosis and herpes zoster, were reported: esophageal candidiasis (*n* = 1) in the upadacitinib 15-mg group and esophageal candidiasis (*n* = 1) in the upadacitinib 30-mg group.

f One patient on upadacitinib 15 mg had an event of gastrointestinal perforation that appeared twice in the table (diverticular perforation and abdominal abscess).

g Malignancies excluding NMSC: malignant melanoma (*n* = 1), bladder transitional cell carcinoma (*n* = 1) in the upadacitinib 15-mg group; malignant fibrous histiocytoma (*n* = 1) in the upadacitinib 30-mg group.

h One patient with deep vein thrombosis in the upadacitinib 30-mg group.

**Table 3.** Treatment-emergent adverse events in the cumulative maintenance population treated with upadacitinib through week 48 of the long-term extension study (up to 285 weeks of exposure), reported as exposure-adjusted event rates.

	Cumulative maintenance population (randomized responders)^b^	
Adverse event, E (E/100 PYs)^a^	Upadacitinib 15 mg *N* = 221; PYs = 350.7 Median exposure = 51.6 weeks Min, max = 1.3, 285.3	Upadacitinib 30 mg *N* = 229; PYs = 432.8 Median exposure = 103.1 weeks Min, max = 1.1, 279.9
Overview of TEAEs		
*Any AE*	993 (283.1)	1183 (273.4)
*Any AE leading to the discontinuation of the study drug*	25 (7.1)	23 (5.3)
*Any AE with a reasonable possibility of being related to the study drug^c^*	253 (72.1)	313 (72.3)
*COVID-19 infection*	36 (10.3)	70 (16.2)
*Severe AE*	57 (16.3)	55 (12.7)
*Serious AE*	56 (16.0)	63 (14.6)
*Deaths*	1 (0.3)^d^	0 (0)
AE of Special Interest		
*Serious infection*	12 (3.4)	19 (4.4)
*Opportunistic infection, excluding tuberculosis and herpes zoster^e^*	2 (0.6)	1 (0.2)
*Active tuberculosis*	0	0
*Herpes zoster*	9 (2.6)	24 (5.5)
*Adjudicated gastrointestinal perforation*	4 (1.1)^f^	1 (0.2)
*Anemia*	19 (5.4)	23 (5.3)
*Neutropenia*	12 (3.4)	11 (2.5)
*Lymphopenia*	18 (5.1)	37 (8.5)
*Creatine phosphokinase elevation*	21 (6.0)	20 (4.6)
*Hepatic disorder*	27 (7.7)	39 (9.0)
*Renal dysfunction*	0	1 (0.2)
*Malignancies excluding NMSC^g^*	3 (0.9)	3 (0.7)
*NMSC*	0	2 (0.5)
*Lymphoma*	0	0
*Adjudicated MACE*	0	0
*Adjudicated VTE*	0	1 (0.2)^h^
*Serious hypersensitivity reactions*	0	0
*Retinal detachments*	0	0

AE, adverse event; COVID-19, coronavirus disease 2019; E, event; LTE, long-term extension; MACE, major adverse cardiovascular event; NMSC, nonmelanoma skin cancer; PY, patient-years; TEAE, treatment-emergent adverse event; VTE, venous thromboembolic event.

a Exposure-adjusted event rates were expressed as the number of events per 100 patient-years (E/100 PYs). TEAEs were defined as any AEs with an onset date on or after the first dose of the study drug in the maintenance period and up to 30 days past the last dose of the study drug in the maintenance or LTE period or until 1 day prior to the rescue in the maintenance or LTE study; cutoff date: 19 December 2023.

b Patients in the cumulative maintenance population (randomized responders) received up to 285 weeks (> 5 years) of upadacitinib maintenance therapy. This population included patients who entered the U-ENDURE double-blind maintenance study (randomized responders) and continued in the LTE on the same maintenance treatment dose.

c As assessed by the study investigator.

d An event of suicide with no reasonable possibility of being related to the study drug as assessed by the investigator.

e Three events of opportunistic infections, excluding tuberculosis and herpes zoster, were reported: esophageal candidiasis and *Pneumocystis jirovecii* pneumonia in the upadacitinib 15-mg group, and esophageal candidiasis in the upadacitinib 30-mg group. The event of *Pneumocystis jirovecii* pneumonia was serious and led to the discontinuation of the study drug.

f One patient on upadacitinib 15 mg had an event of gastrointestinal perforation that appeared twice in the table (diverticular perforation and abdominal abscess).

g Malignancies excluding NMSC: ovarian cancer metastatic (*n* = 1), malignant melanoma (*n* = 1), bladder transitional cell carcinoma (*n* = 1) in the upadacitinib 15-mg group; adenocarcinoma of colon (*n* = 1), invasive lobular breast carcinoma (*n* = 1), and malignant fibrous histiocytoma (*n* = 1) in the upadacitinib 30-mg group.

h One patient with deep vein thrombosis in the upadacitinib 30-mg group.

Among patients with 48-week matured data in the LTE study, event rates for any AEs (upadacitinib 15 mg, 226.9; upadacitinib 30 mg, 241.0 E/100 PY), serious AEs (upadacitinib 15 mg, 9.6; upadacitinib 30 mg, 10.3 E/100 PY), severe AEs (upadacitinib 15 mg, 9.6; upadacitinib 30 mg, 9.0 E/100 PY), and AEs leading to discontinuation (upadacitinib 15 mg, 3.0; upadacitinib 30 mg, 4.0 E/100 PY) were similar among patients treated with either upadacitinib dose. Event rates for most AEs of special interest were similar among upadacitinib-treated groups, except for numerically lower rates of herpes zoster (1.5, 4.7 E/100 PY), lymphopenia (6.1, 9.0 E/100 PY), and hepatic disorders (6.6, 9.0 E/100 PY) among upadacitinib 15-mg-treated patients (Table 2). Serious infections occurred at rates of 1.5 and 2.5 E/100 PY for upadacitinib 15-mg and upadacitinib 30-mg-treated patients, respectively. No occurrences of adjudicated GI perforation were reported in patients treated with upadacitinib 30 mg; 3 events in 2 patients were reported in upadacitinib 15-mg-treated patients (1.5 E/100 PY). One death (EAER of 0.5 E/100 PY) by suicide was reported in a patient on upadacitinib 15 mg.

The rate of NMSC was 0.9 E/100 PY on upadacitinib 30 mg dose, and no such events were reported on the 15 mg dose. Three patients developed malignancies other than NMSC. Two patients (2 AEs, 1.0 E/100 PY) were treated with upadacitinib 15 mg who developed respectively, malignant melanoma [reasonable possibility of being related to study drug as deemed by the investigator] and transitional cell carcinoma of the urothelial bladder [no reasonable possibility of being related to study drug as deemed by the investigator]). One patient (1 AE, 0.3 E/100 PY) treated with upadacitinib 30 mg experienced malignant fibrous histiocytoma [assessed by investigator as having a reasonable possibility of being related to the study drug]. A VTE was reported in 1 patient (0.3 E/100 PY) treated with upadacitinib 30 mg.

Additionally, safety was analyzed for the cumulative maintenance population, which included both the 52-week maintenance and LTE study patients, with more than 5 years of exposure. In this analysis, event rates for any AE, serious AE, and severe AE were overall similar in the 2 upadacitinib dose regimens (Table 3). The cumulative rate of serious infections across maintenance and LTE was also similar in patients treated with either upadacitinib 15 mg or 30 mg, at 3.4 and 4.4 E/100 PY, respectively. Herpes zoster was reported at a slightly lower rate (2.6 E/100 PY) in patients treated with upadacitinib 15 mg as compared to 30 mg (5.5 E/100 PY); similar results were observed for lymphopenia (5.1 E/100 PY and 8.5 E/100 PY). The adjudicated VTE rate was 0.2 E/100 PY, which was contributed by 1 LTE patient treated with upadacitinib 30 mg as described above; no such events occurred in patients treated with the 15 mg dose. The rate of NMSC was 0.5 E/100PY in patients treated with upadacitinib 30 mg and no such events were reported in patients treated with 15 mg. Malignancies excluding NMSC were reported for 3 patients (3 AEs, 0.9 E/100 PY) treated with upadacitinib 15 mg and 3 patients (3 AEs, 0.7 E/100 PY) treated with upadacitinib 30 mg during the 52-week maintenance period, without a trend noted in the type of malignancy, as previously reported.^13^ Of these malignancies, excluding NMSC, there were 3 that were not reasonably related to the study drug: ovarian metastatic cancer (received upadacitinib 15 mg); invasive lobular breast carcinoma, and colon adenocarcinoma (both received upadacitinib 30 mg).^8^ Three other malignancies, excluding NMSC (2 in patients treated with upadacitinib 15 mg and 1 in those treated with upadacitinib 30 mg), were reported during the LTE 48 weeks, as described above. Other AEs of special interest were reported at a similar rate among patients treated with upadacitinib 15 and 30 mg. No serious hypersensitivity reactions, retinal detachments, active tuberculosis, lymphoma, or adjudicated MACE occurred in patients during the LTE or maintenance studies. Incidence rates were reported for the cumulative maintenance population through LTE week 48 (Table S3). The most common AEs of special interest (≥5.0 events/100 PY) were hepatic disorder, lymphopenia, creatine phosphokinase elevation, herpes zoster, and anemia.

In both the LTE only and cumulative safety populations, herpes zoster infections were all nonserious, most involved a single dermatome, with 1 patient having 3 dermatome involvement (Table 3). All but 1 herpes zoster event were mild to moderate, with the majority reported as reasonably related to the study drug. There were no cases of disseminated herpes zoster and no events of central nervous system, lung, or liver involvement. There was 1 event of ophthalmic herpes zoster in the upadacitinib 30-mg-treatment group among randomized responders in the cumulative safety population. Two out of all patients on upadacitinib treatment who developed herpes zoster during the study were vaccinated with the herpes zoster vaccine; others were not vaccinated.

## 4. Discussion

This interim analysis of the U-ENDURE LTE study evaluated efficacy and safety for patients with moderately to severely active CD treated with upadacitinib. Sustained efficacy was observed for clinical, endoscopic, HRQoL, and inflammatory biomarker outcomes, with over 75% of patients sustaining clinical remission throughout the LTE study (per AO analysis).

According to STRIDE-II, symptomatic remission and normalization of hs-CRP and FCP are intermediate treatment targets, while endoscopic healing, normalization of QoL, and absence of a disability serve as long-term targets.^14^ Normalized hs-CRP and FCP values during induction have been associated with improvements in long-term clinical outcomes in anti-TNF-treated patients.^15-17^ In this analysis, upadacitinib treatment demonstrated the achievement of intermediate and long-term goals recommended by STRIDE-II, including high rates for clinical, endoscopic, HRQoL outcomes, and biomarker normalization. High proportions of patients treated with either the upadacitinib 15 mg or 30 mg maintenance regimen achieved clinical remission throughout the LTE study, with the primary reason for patient discontinuation being other (nonspecified) reasons unrelated to the study drug. Durability of response was achieved for all clinical outcomes, with high rates of patients sustaining clinical remission through LTE week 48. Treating to target endoscopic healing is associated with improved long-term outcomes and may reduce the risk of bowel damage.^18^ In this study, patients attained robust rates for long-term endoscopic outcomes after 2 years of upadacitinib maintenance therapy. Additionally, upadacitinib-treated patients sustained long-term HRQoL improvements through LTE week 48, indicating that QoL improvements were generally sustained after 2 years of maintenance treatment.

Clinical and endoscopic assessments do not fully capture the burden of disease from a patient perspective, and QoL measurements are often used to provide important insights into the influence of CD on patients’ lives and their professional and personal productivity. Patients with CD often have debilitating symptoms that affect their physical, psychological, and social-emotional well-being. Previous studies have shown that CD disease activity is directly correlated with IBDQ score and other HRQoL outcomes, as patients in remission have shown improvements in HRQoL outcomes.^19^^,^^20^ Multiple aspects of HRQoL have been captured during the 48-week LTE treatment period. Overall, stable HRQoL outcomes were maintained throughout the LTE with upadacitinib treatment, as indicated by consistent scores across IBDQ, FACIT-F, SF-36v2 PCS and MCS, WPAI-CD absenteeism and presenteeism, and WPAI-CD overall work and activity impairment. These outcomes enable healthcare providers to better understand the long-term impact of upadacitinib on several aspects of HRQoL, which are important to patients.

The effect of treatment escalation with open-label upadacitinib 30 mg among patients who responded to upadacitinib induction therapy but experienced loss of response to upadacitinib 15 mg or placebo during 52-week maintenance or LTE treatment was reported previously.^21^ Similar to the previously reported rescue treatment outcomes during maintenance, patients who experienced loss of response to upadacitinib 15 mg during LTE treatment, most regained clinical response, and many achieved clinical remission 12 weeks after escalating to upadacitinib 30 mg therapy and were able to remain in the study.^21^ Clinical improvements after escalating to upadacitinib 30 mg therapy were sustained through 24 weeks and were accompanied by improvements in hs-CRP and FCP biomarkers (Figure S6). The results of this post hoc analysis suggest that escalation of treatment from upadacitinib 15 mg to upadacitinib 30 mg may be beneficial to patients with moderately to severely active CD experiencing inadequate response to or interruption in upadacitinib maintenance treatment, and may be an effective option to recapture response in these patients.

Hospitalization and surgery remain relatively common among patients with CD despite improvements in treatment.^22^^,^^23^ CD-related hospitalizations and surgeries may be disruptive to patients’ lives and carry a higher burden, impacting patients’ physical and mental well-being, encompassing potential complications, long-term effects, and financial strain of treatment.^24^ The rates of CD-related hospitalizations/surgeries reported for both upadacitinib doses were low, but the small number of patients with these events limits our ability to make conclusions.

Understanding the long-term efficacy and safety of medicines is critical for chronic diseases like CD to support evidence-based treatment decisions. Safety outcomes in this study were evaluated in patients who received a minimum of 2 years of upadacitinib treatment. No new safety signals were observed, and the safety profile was consistent with that of the phase 2 open-label extension study (CELEST) and the phase 3 primary maintenance population.^8^^,^^25^

During the LTE study, 3 malignancies, excluding NMSC, were reported. These patients were randomized responders and included in both LTE and cumulative maintenance populations (Tables 2 and 3). No pattern or cluster of malignancies, excluding NMSC, was observed with upadacitinib use in patients with CD. This observation was similar to those for other rheumatoid arthritis, psoriatic arthritis, and atopic dermatitis indications.^26^ Further evaluation of the risk of malignancy in patients with CD or ulcerative colitis is ongoing in a cohort study of long-term safety of upadacitinib in a real-world setting in Europe and is described in the Swiss Summary of the Risk Management Plans.^26^ One death unrelated to the study drug was reported in a patient who committed suicide on upadacitinib 15 mg. No MACE was reported; and 1 VTE event occurred in a patient on upadacitinib 30 mg. These patients were also randomized responders and included in both Tables 2 and 3. Fewer herpes zoster events were observed in upadacitinib 15-mg-treated patients compared with those who received upadacitinib 30 mg during maintenance or the LTE study. Due to low patient numbers (*n* = 6, upadacitinib 15 mg, *n* = 3, upadacitinib 30 mg), the rates of TEAEs could not be analyzed in patients ≥ 65 years of age. Further analysis will be performed upon completion of the LTE to further identify the occurrences of rare, low-incidence, or long-latency TEAEs, including in high-risk subpopulations.

Limitations included the open-label and exploratory nature of the LTE study. As the patients were not randomized in the presented analyses, statistical comparisons could not be made between upadacitinib and placebo treatment groups, and multiplicity control was not applicable. Regardless, the number of patients remaining on placebo in LTE was small due to either rescue of these patients with upadacitinib 30 mg or discontinuation from the study.

Results from this study based on AO analysis should be interpreted with caution, as AO analysis does not account for rescue interventions done for patients who had an inadequate response or were intolerant to therapy during the study and, by definition, was conducted among patients who did not prematurely end study participation at the time of analysis. Although AO analysis can potentially be over-optimistic, our NRI approach was a relatively conservative analysis among all treated patients, accounting for rescue interventions, and where any missing data were assumed to be a nonresponse, essentially treating all missing values as failures. The NRI approach mostly supported observations from the AO approach, supporting the robustness of our long-term efficacy conclusions of upadacitinib 15 mg and upadacitinib 30 mg. Per study design, clinical nonresponders or patients who lost the response to placebo or upadacitinib treatment could be rescued with upadacitinib 30 mg. As a result, a substantial number of patients on placebo and upadacitinib 15 mg were rescued with upadacitinib 30 mg and went from the originally assigned treatment group to the rescue group that was analyzed separately. That could explain the relatively small number of patients included in the efficacy population. While subgroup analysis was also performed on the TNF-IR patient population who met the US FDA label criteria and the results were consistent with the total study population, they should be interpreted with caution, as patient numbers were limited for some subgroups.

In conclusion, this publication demonstrated a favorable risk-benefit of upadacitinib 15 mg and 30 mg QD in patients with moderate-to-severe CD who received at least 2 years of maintenance therapy. Patients were able to safely maintain clinical remission and endoscopic response and sustained improvements in clinical, endoscopic, and HRQoL outcomes, demonstrating the durability of upadacitinib efficacy with no new safety signals identified.

## Supplementary Material

jjaf138_Supplementary_Data

## Data Availability

AbbVie is committed to responsible data sharing regarding the clinical trials we sponsor. This includes access to anonymized, individual, and trial-level data (analysis data sets), as well as other information (eg, protocols, clinical study reports, or analysis plans), as long as the trials are not part of an ongoing or planned regulatory submission. This includes requests for clinical trial data for unlicensed products and indications. These clinical trial data can be requested by any qualified researchers who engage in rigorous, independent, scientific research, and will be provided following review and approval of a research proposal, Statistical Analysis Plan (SAP), and execution of a Data Sharing Agreement (DSA). Data requests can be submitted at any time after approval in the US and Europe and after acceptance of this manuscript for publication. The data will be accessible for 12 months, with possible extensions considered. For more information on the process or to submit a request, visit the following link: https://vivli.org/ourmember/abbvie/ and then select “Home.”
