# Supplementary material for: Efficacy and safety of upadacitinib maintenance therapy in patients with moderately to severely active Crohn’s disease: 2-year results from the U-ENDURE Long-Term Extension study
Source: J Crohns Colitis. 2025 Jul 24;19(8):jjaf138. doi: 10.1093/ecco-jcc/jjaf138 (PMC12459986; doi:10.1093/ecco-jcc/jjaf138)
Supplement: jjaf138_Supplementary_Data [file jjaf138_supplementary_data.zip › Supp footnotes_UPA CD LTE 2yr.docx]

**SUPPLEMENTARY MATERIAL**

**Efficacy and Safety of Upadacitinib Maintenance Treatment in Patients With Moderately to Severely Active Crohn’s Disease: 2-Year Results From the U-ENDURE Long-Term Extension Study**

Short title: Upadacitinib CD Long-Term Extension: 2-Year Results (46/50 characters)

Edward V. Loftus Jr,^a^ Geert D’Haens,^b^ Edouard Louis,^c^ Miguel Regueiro,^d^ Vipul Jairath,^e^ Fernando Magro,^f,g,h^ Hiroshi Nakase,^i^ Elena Dubcenco,^j^ Ana Paula Lacerda,^j^ Sharanya Ford,^j^ Tian Feng,^j^ Benjamin Duncan,^j^ Irina Fish,^j^ Colla Cunneen^,j^ Samuel I. Anyanwu,^j^ Fernando Aponte,^j^ Jenny Griffith,^j^ Irina Blumenstein^k^

*^a^Division of Gastroenterology and Hepatology, Mayo Clinic College of Medicine and Science, Rochester, MN, USA*

*^b^Department of Gastroenterology and Hepatology, Amsterdam University Medical Centre, Amsterdam, The Netherlands*

*^c^Department of Hepato-Gastroenterology and Digestive Oncology, University Hospital CHU of Liège, Liège, Belgium*

*^d^Department of Gastroenterology and Hepatology, Cleveland Clinic Foundation, Cleveland, OH, USA*

*^e^Department of Medicine, Western University, London, ON, Canada*

*^f^Unit of Pharmacology and Therapeutics, Department of Biomedicine and Medicine, Porto, Portugal*

^g^Department *of Gastroenterology, São João University Hospital Center, Porto, Portugal*

*^h^CINTESIS@RISE, Department of Medicine, University of Porto, Porto, Portugal*

*^i^Department of Gastroenterology and Hepatology, Sapporo Medical University School of Medicine, Sapporo, Japan*

*^j^AbbVie, Inc, North Chicago, IL, USA*

*^k^Goethe University Frankfurt, University Hospital, Department of Gastroenterology, Hepatology and Clinical Nutrition Frankfurt, Germany*

**Supplementaty Footnotes**

**Figure S1. Disposition for the Efficacy Population in the Upadacitinib U-ENDURE Long-Term Extension Study.**

LTE, long-term extension

**Figure S2. Disposition for the Safety Populations in the Upadacitinib U-ENDURE Long-Term Extension Study.**

LTE, long-term extension; OL, open-label

^a^The cumulative safety population included patients who entered the U-ENDURE double-blind maintenance study (randomized responders) and continued in the LTE on the same maintenance treatment dose of upadacitinib 15 mg or upadacitinib 30 mg, with maximum exposure to upadacitinib at 285 weeks (N=450; upadacitinib 15 mg, median exposure=51.6 weeks; upadacitinib 30 mg, median exposure=103.1 weeks).

^b^The long-term extension only safety population included patients on upadacitinib 15 mg or upadacitinib 30 mg in the LTE (patients from U-ENDURE efficacy population and U-ENDURE other population, with maximum exposure at 229 weeks, N=280; upadacitinib 15 mg, median exposure=94.3 weeks; upadacitinib 30 mg, median exposure=91.3 weeks).

**Figure S3. Reasons for Study Discontinuation in the U-ENDURE Long-Term Extension Study.**

CD, Crohn’s disease; LTE, long-term extension.

**Figure S4. Clinical and Endoscopic Outcomes for Patients Who Met the US FDA Label Criteria and Were Treated With Upadacitinib Through Week 48 of the U-ENDURE Long-Term Extension Study.**

AO, as-observed; CDAI, Crohn’s disease activity index; CI, confidence interval; CR-100, clinical response-100; LTE, long-term extension; NRI, nonresponder imputation; QD, once-daily; TNF, tumor necrosis factor; US FDA, United States Food and Drug Administration.

US FDA label criteria: Patients who had an inadequate response or intolerance to one or more prior TNF inhibitors, had a CDAI score ≥ 220 at induction baseline, and achieved CR-100 after 12 weeks of upadacitinib 45 mg QD induction treatment. AO analysis used all available data up to the initiation of open-label upadacitinib 30 mg rescue therapy and did not impute values for missing data. For NRI analysis on binary variables, patients were categorized as nonresponder after initiation of any protocol rescue medications or missing data.

**Figure S5. Mean Change From Baseline of Induction For SF-36v2 and WPAI-CD in Patients Treated With Upadacitinib Through Week 48 of the U-ENDURE Long-Term Extension Study.**

AO, as-observed; CI, confidence intervals; LTE, long-term extension; MCS, Mental Component Score; NRI, nonresponder imputation; PCS, physical component score; SF-36v2, Short-Form Health Survey-36 version 2; WPAI-CD, Work Productivity and Activity Impairment-Crohn’s disease.

Baseline was defined as week 0 of induction. AO analysis used all available data up to the initiation of open-label upadacitinib 30 mg rescue therapy and did not impute values for missing data. For NRI analysis on binary variables, patients were categorized as nonresponder after initiation of any protocol rescue medications or missing data.

**Figure S6. Clinical and Inflammatory Marker Endpoints for Patients Who Received Rescue Therapy With Open-label Upadacitinib 30 mg Through For 24 Weeks in the U-ENDURE Long-Term Extension Study.**

AO, observed; CDAI, Crohn’s disease activity index; CI, confidence interval; hs-CRP, high-sensitivity C-reactive protein; FCP, fecal calprotectin; LTE, long-term extension; SF/APS, stool frequency/abdominal pain score.

Baseline refers to baseline (week 0) of induction. A-D) Shown are the percentages of responding patients for each endpoint. E and F) Shown are the absolute mean values for hs-CRP or FCP for patients in each treatment group. AO analysis was used for all available measurements and did not impute values for missing data. 95% CI for the response rate was based on the normal approximation to the binomial distribution.
