# Supplementary material for: Efficacy and safety of upadacitinib maintenance therapy in patients with moderately to severely active Crohn’s disease: 2-year results from the U-ENDURE Long-Term Extension study
Source: J Crohns Colitis. 2025 Jul 24;19(8):jjaf138. doi: 10.1093/ecco-jcc/jjaf138 (PMC12459986; doi:10.1093/ecco-jcc/jjaf138)
Supplement: jjaf138_Supplementary_Data [file jjaf138_supplementary_data.zip › Table S1.docx]

**Table S1. Demographics and Clinical Characteristics of Patients in the U-ENDURE Long-Term Extension Study at Baseline of Induction.**

| Characteristic, n (%) unless otherwise noted | Upadacitinib 15 mg  N = 107 | Upadacitinib 30 mg  N = 137 |
| --- | --- | --- |
| Sex |  |  |
| *Female*  *Male* | 42 (39.3)  65 (60.7) | 53 (38.7)  84 (61.3) |
| Age, |  |  |
| *Mean (SD), years* | 38.7 (14.6) | 34.6 (12.1) |
| *18 - < 40* | 61 (57.0) | 95 (69.3) |
| *40 - < 65* | 40 (37.4) | 39 (28.5) |
| *≥ 65* | 6 (5.6) | 3 (2.2) |
| Body mass index, kg/m^2^, mean (SD) | 23.9 (6.0) | 23.9 (6.0) |
| Race |  |  |
| *American Indian or Alaska Native* | 0 | 0 |
| *Asian* | 25 (23.4) | 40 (29.2) |
| *Black or African American* | 1 (0.9) | 4 (2.9) |
| *Native Hawaiian or Other Pacific Islander* | 0 | 0 |
| *White* | 80 (74.8) | 91 (66.4) |
| *Multiple* | 1 (0.9) | 2 (1.5) |
| Ethnicity |  |  |
| *Hispanic or Latino* | 11 (10.3) | 8 (5.8) |
| *Not Hispanic* | 96 (89.7) | 129 (94.2) |
| Tobacco/nicotine use |  |  |
| *Current* | 13 (12.1) | 26 (19.0) |
| *Former* | 19 (17.8) | 19 (13.9) |
| *Never* | 75 (70.1) | 91 (66.4) |
| *Unknown* | 0 | 1 (0.7) |
| CDAI, mean (SD) | N = 106  296 (83) | N = 137  309 (76) |
| SES-CD, mean (SD) | 15.7 (7.2) | 14.9 (7.9) |
| Average daily very soft or liquid SF, mean (SD) | N = 106  5.1 (2.3) | N = 137  5.2 (2.7) |
| Average daily APS, mean (SD) | N = 106  1.8 (0.7) | N = 137  2.0 (0.6) |
| Disease duration |  |  |
| *Mean (SD), years* | 10.3 (9.3) | 7.7 (6.2) |
| *≤ 5* | 39 (36.4) | 55 (40.1) |
| *> 5* | 68 (63.6) | 82 (59.9) |
| Disease location |  |  |
| *Ileal Only* | 6 (5.6) | 14 (10.2) |
| *Colonic Only* | 52 (48.6) | 61 (44.5) |
| *Ileal-Colonic* | 49 (45.8) | 62 (45.3) |
| hs-CRP, mg/L,  *Median (range)*  *Median, Q1, Q3, IQR* | N = 105  10.2 (0.2, 110.0)  N=105  10.2 (2.6, 21.8, 19.3) | N = 133  8.9 (0.2, 124.0)  N=133  8.9 (4.4, 22.4, 18.0) |
| FCP, mg/kg,  *Median (range)*  *Median, Q1, Q3, IQR* | N = 95  1700 (30, 28 800)  N=95  1700 (490, 3187, 2697) | N = 122  1554 (30, 28 800)  N=122  1554 (521, 3262, 2741) |
| Medications at baseline |  |  |
| *Immunosuppressant use* | 5 (4.7) | 3 (2.2) |
| *Aminosalicylates use* | 29 (27.1) | 31 (22.6) |
| *Corticosteroid use* | 39 (36.4) | 48 (35.0) |
| Biologic therapy - IR | 70 (65.4) | 96 (70.1) |
| *Prior failure to anti-TNF* | N = 70  66 (94.3) | N = 96  92 (95.8) |
| Nonbiologic therapy - IR | 37 (34.6) | 41 (29.9) |
| Number of prior failed biologic therapies | N = 70 | N = 96 |
| *1* | 31 (44.3) | 43 (44.8) |
| *2* | 20 (28.6) | 32 (33.3) |
| *> 2* | 19 (27.1) | 21 (21.9) |

APS, abdominal pain score; CDAI, Crohn’s disease activity index; FCP, fecal calprotectin; hs-CRP, high-sensitivity C-reactive protein; IR, inadequate response; SD, standard deviation; SES-CD, SF, stool frequency; Simple Endoscopic Score for Crohn’s Disease; TNF, tumor necrosis factor.

Percentages were calculated on nonmissing values. Baseline was defined as week 0 of induction.
