# Supplementary material for: Efficacy and safety of upadacitinib maintenance therapy in patients with moderately to severely active Crohn’s disease: 2-year results from the U-ENDURE Long-Term Extension study
Source: J Crohns Colitis. 2025 Jul 24;19(8):jjaf138. doi: 10.1093/ecco-jcc/jjaf138 (PMC12459986; doi:10.1093/ecco-jcc/jjaf138)
Supplement: jjaf138_Supplementary_Data [file jjaf138_supplementary_data.zip › Table S2.docx]

**Table S2. Incidence Rates For CD-Related Hospitalizations and CD-Related Surgeries In Upadacitinib-Treated Patients Through LTE Week 48**

| **Variable, n/PYs (n/100 PY)^a^** | **Upadacitinib 15 mg**  **N=107** | **Upadacitinib 30 mg**  **N=137** |
| --- | --- | --- |
| CD-related hospitalizations | 3/198.7(1.5) | 8/258.1 (3.1) |
| CD-related surgeries | 3/199.1 (1.5) | 3/253.5 (1.2) |

CD, Crohn’s disease; LTE, long-term extension; PY, patient-years; QD, once-daily.

^a^All available measurements before initiation of open-label upadacitinib 30 mg QD rescue were used for

analysis, and AO analysis did not impute values for missing data.
