# Supplementary material for: Efficacy and safety of upadacitinib maintenance therapy in patients with moderately to severely active Crohn’s disease: 2-year results from the U-ENDURE Long-Term Extension study
Source: J Crohns Colitis. 2025 Jul 24;19(8):jjaf138. doi: 10.1093/ecco-jcc/jjaf138 (PMC12459986; doi:10.1093/ecco-jcc/jjaf138)
Supplement: jjaf138_Supplementary_Data [file jjaf138_supplementary_data.zip › Table S3.docx]

**Table S3. Treatment-Emergent Adverse Events in the Cumulative Maintenance Population Treated With Upadacitinib Through Week 48 of the Long-Term Extension Study (Up to 285 Weeks of Exposure), Reported as Exposure-Adjusted Incidence Rates.**

|  | **Cumulative Maintenance Population (Randomized Responders)^b^** | |
| --- | --- | --- |
| **AE of Special Interest, n/PYs (n/100 PY)^a^** | **Upadacitinib 15 mg**  **N = 221**  **Median exposure = 51.6 weeks**  **Min, max = 1.3, 285.3 weeks** | **Upadacitinib 30 mg  N = 229**  **Median exposure = 103.1 weeks**  **Min, max = 1.1, 279.9 weeks** |
| Serious infection | 10/343.7 (2.9) | 16/405.8 (3.9) |
| Opportunistic infection, excluding tuberculosis and herpes zoster^c^ | 2/349.4 (0.6) | 1/432.6 (0.2) |
| Active tuberculosis | 0/350.7 | 0/432.8 |
| Herpes zoster | 9/337.1 (2.7) | 22/394.3 (5.6) |
| Adjudicated gastrointestinal perforation^d^ | 3/350.7 (0.9) | 1/432.8 (0.2) |
| Anemia | 15/336.7 (4.5) | 20/402.8 (5.0) |
| Neutropenia | 5/340.8 (1.5) | 9/423.0 (2.1) |
| Lymphopenia | 12/328.4 (3.7) | 20/392.3 (5.1) |
| Creatine phosphokinase elevation | 14/330.7 (4.2) | 16/409.6 (3.9) |
| Hepatic disorder | 12/327.5 (3.7) | 22/401.1 (5.5) |
| Renal dysfunction | 0/350.7 | 1/429.8 (0.2) |
| Malignancies excluding NMSC^e^ | 3/350.5 (0.9) | 3/432.7 (0.7) |
| NMSC | 0/350.7 | 2/429.3 (0.5) |
| Lymphoma | 0/350.7 | 0/432.8 |
| Adjudicated MACE | 0/350.7 | 0/432.8 |
| Adjudicated VTE | 0/350.7 | 1/432.7 (0.2)^f^ |
| Serious hypersensitivity reactions | 0/350.7 | 0/432.8 |
| Retinal detachments | 0/350.7 | 0/432.8 |

AE, adverse event; LTE, long-term extension; MACE, major adverse cardiovascular event; n, number; NMSC, nonmelanoma skin cancer; PY, patient-year; TEAE, treatment-emergent adverse event; VTE, venous thromboembolic events.

^a^Exposure-adjusted incidence rates were expressed as the number of patients (n)/100 PY. TEAEs were defined as any AEs with an onset date on or after the first dose of the study drug in the maintenance period and up to 30 days past the last dose of the study drug in the maintenance or LTE period; cutoff date 19 December 2023.

^b^Patients in the cumulative maintenance population (randomized responders) received up to 285 weeks (> 5 years) of upadacitinib maintenance therapy. This population included patients who entered the U-ENDURE double-blind maintenance study (randomized responders) and continued in the LTE on the same maintenance treatment dose.

^c^Three events of opportunistic infections, excluding tuberculosis and herpes zoster, were reported: esophageal candidiasis and *Pneumocystis jirovecii* pneumonia in the upadacitinib 15 mg group, and esophageal candidiasis in the upadacitinib 30 mg group. The event of *Pneumocystis jirovecii* pneumonia was serious and led to the discontinuation of the study drug.

^d^One patient on upadacitinib 15 mg had an event of gastrointestinal perforation that appeared twice in the table (diverticular perforation and abdominal abscess).

^e^Malignancies excluding NMSC: ovarian cancer metastatic (n = 1), malignant melanoma (n = 1), bladder transitional cell carcinoma (n = 1) in the upadacitinib 15 mg group; adenocarcinoma of colon (n = 1), invasive lobular breast carcinoma (n = 1), and malignant fibrous histiocytoma (n = 1) in the upadacitinib 30 mg group.

^f^One patient with deep vein thrombosis in the upadacitinib 30 mg group.
