# Supplementary figures and images for: Efficacy and safety of upadacitinib maintenance therapy in patients with moderately to severely active Crohn’s disease: 2-year results from the U-ENDURE Long-Term Extension study
Source: J Crohns Colitis. 2025 Jul 24;19(8):jjaf138. doi: 10.1093/ecco-jcc/jjaf138 (PMC12459986; doi:10.1093/ecco-jcc/jjaf138)

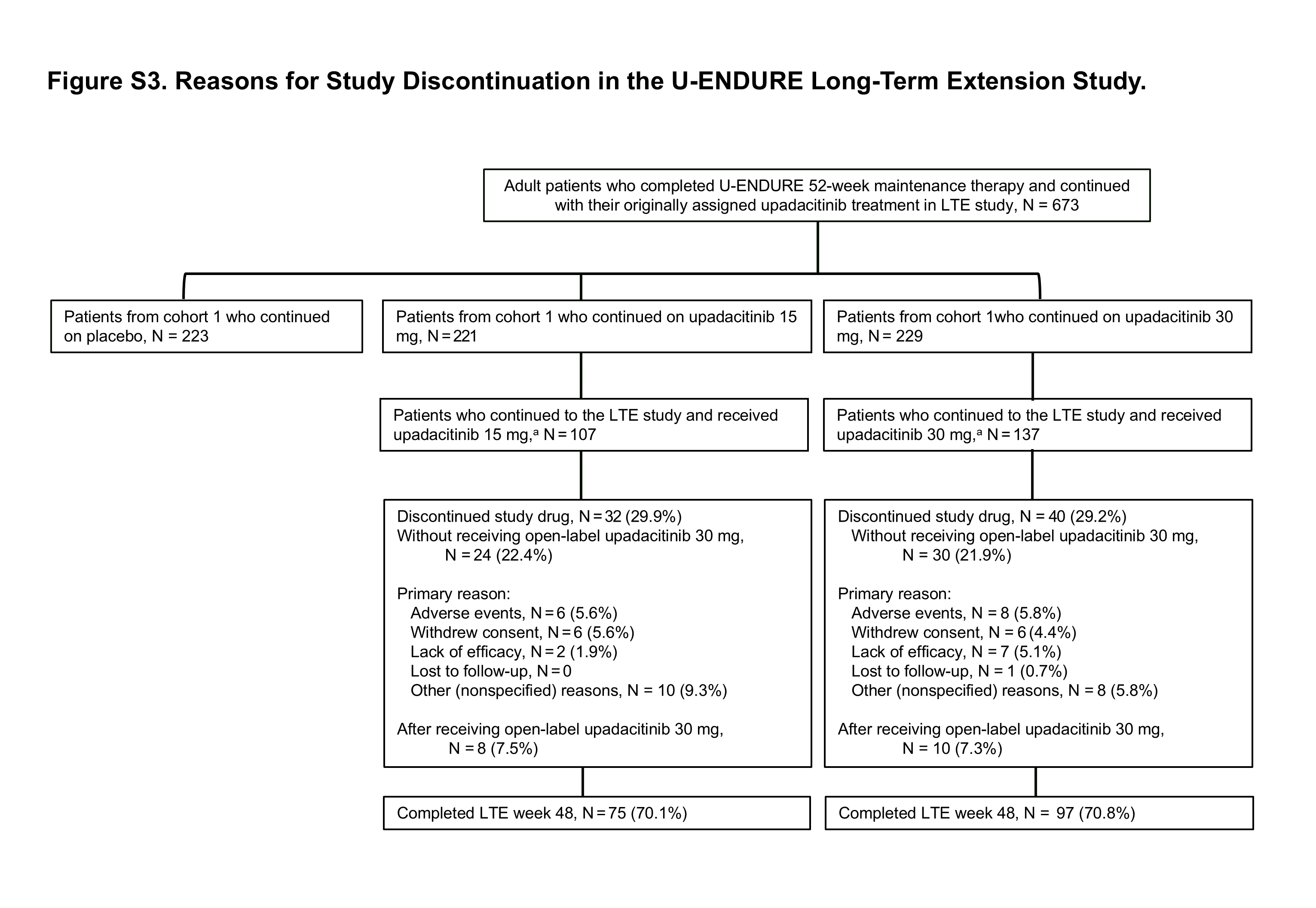

Supplement: jjaf138_Supplementary_Data [file jjaf138_supplementary_data.zip › Figure S3.tif]

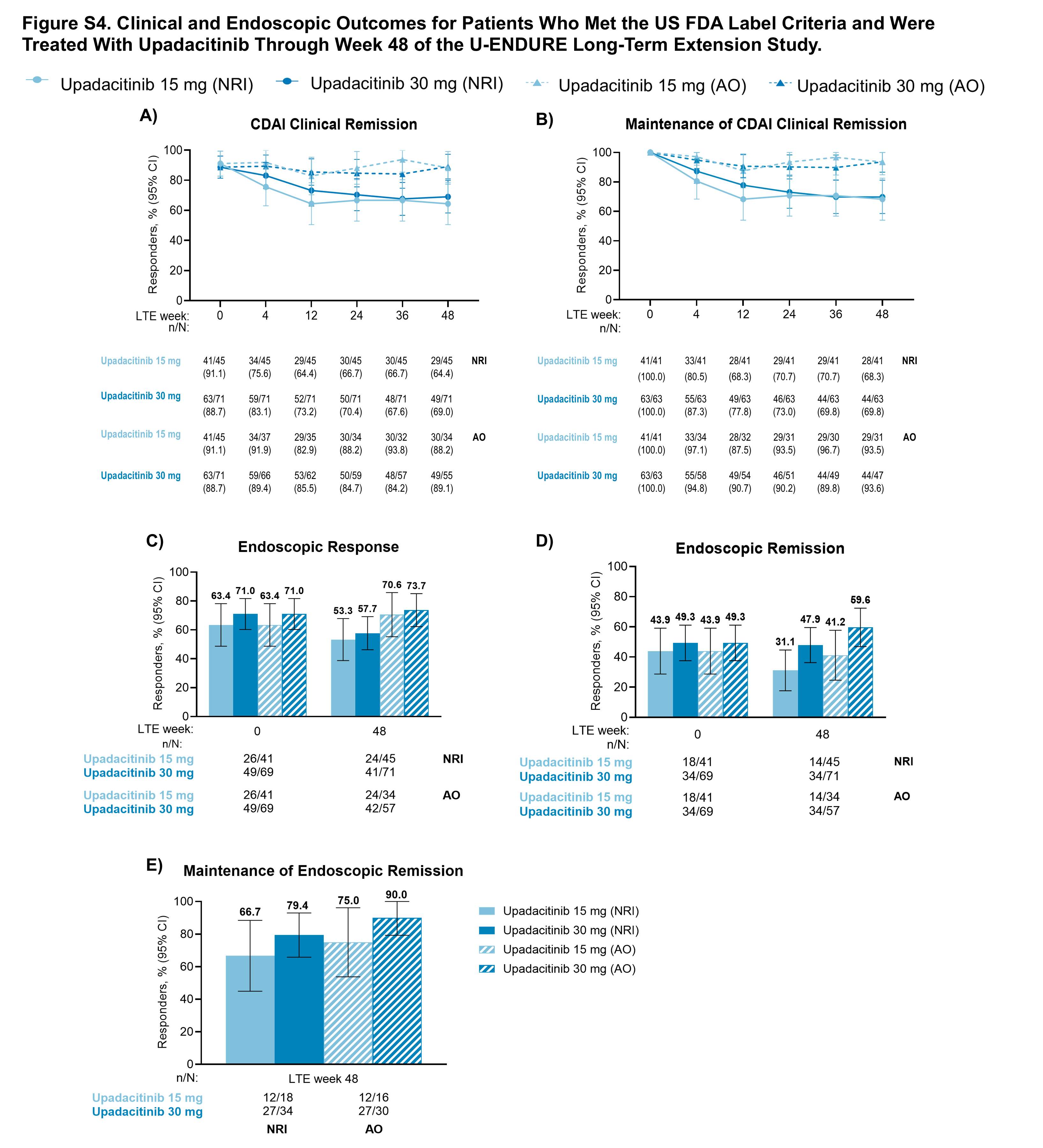

Supplement: jjaf138_Supplementary_Data [file jjaf138_supplementary_data.zip › Figure S4.TIF]

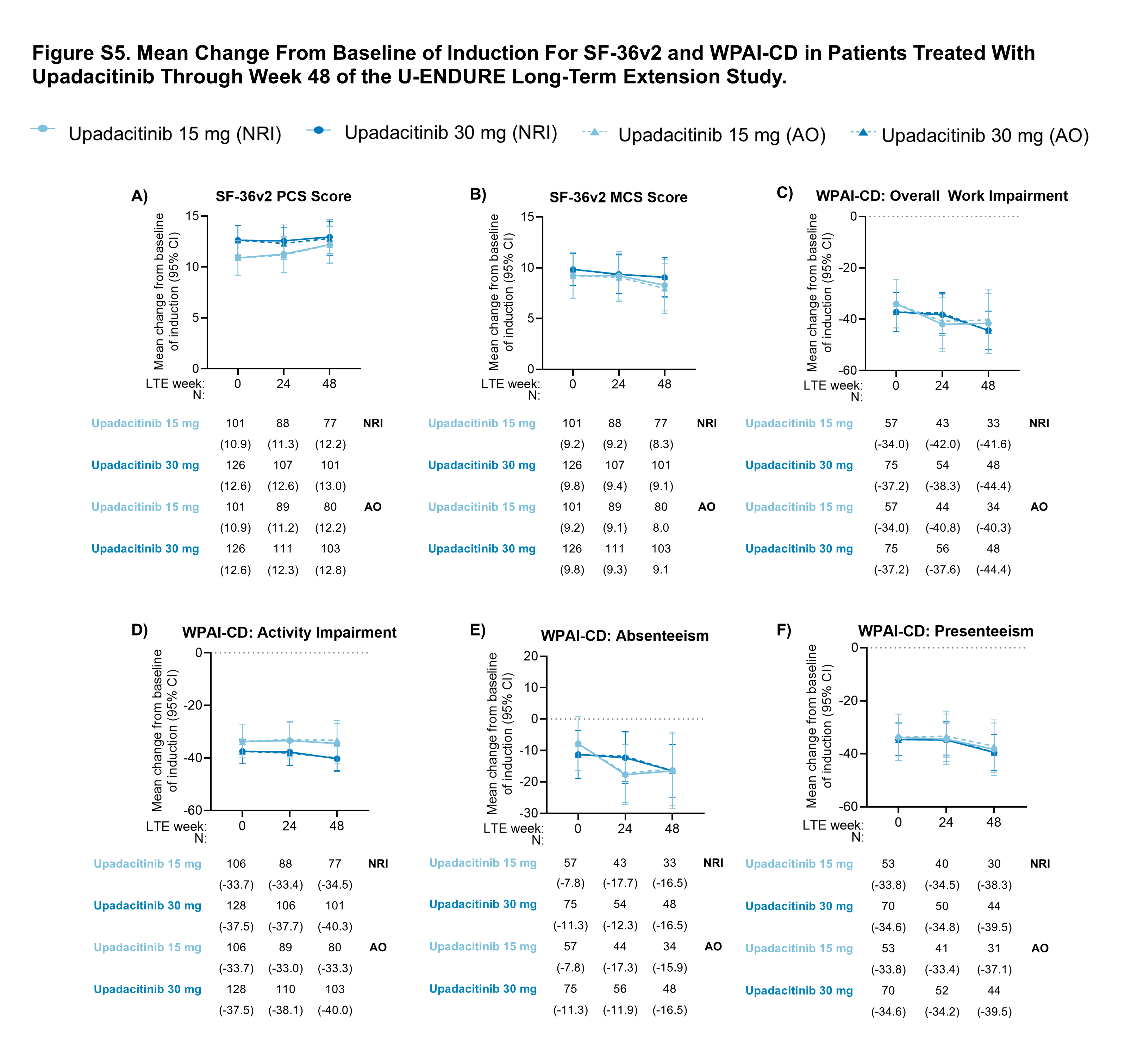

Supplement: jjaf138_Supplementary_Data [file jjaf138_supplementary_data.zip › Figure S5.TIF]

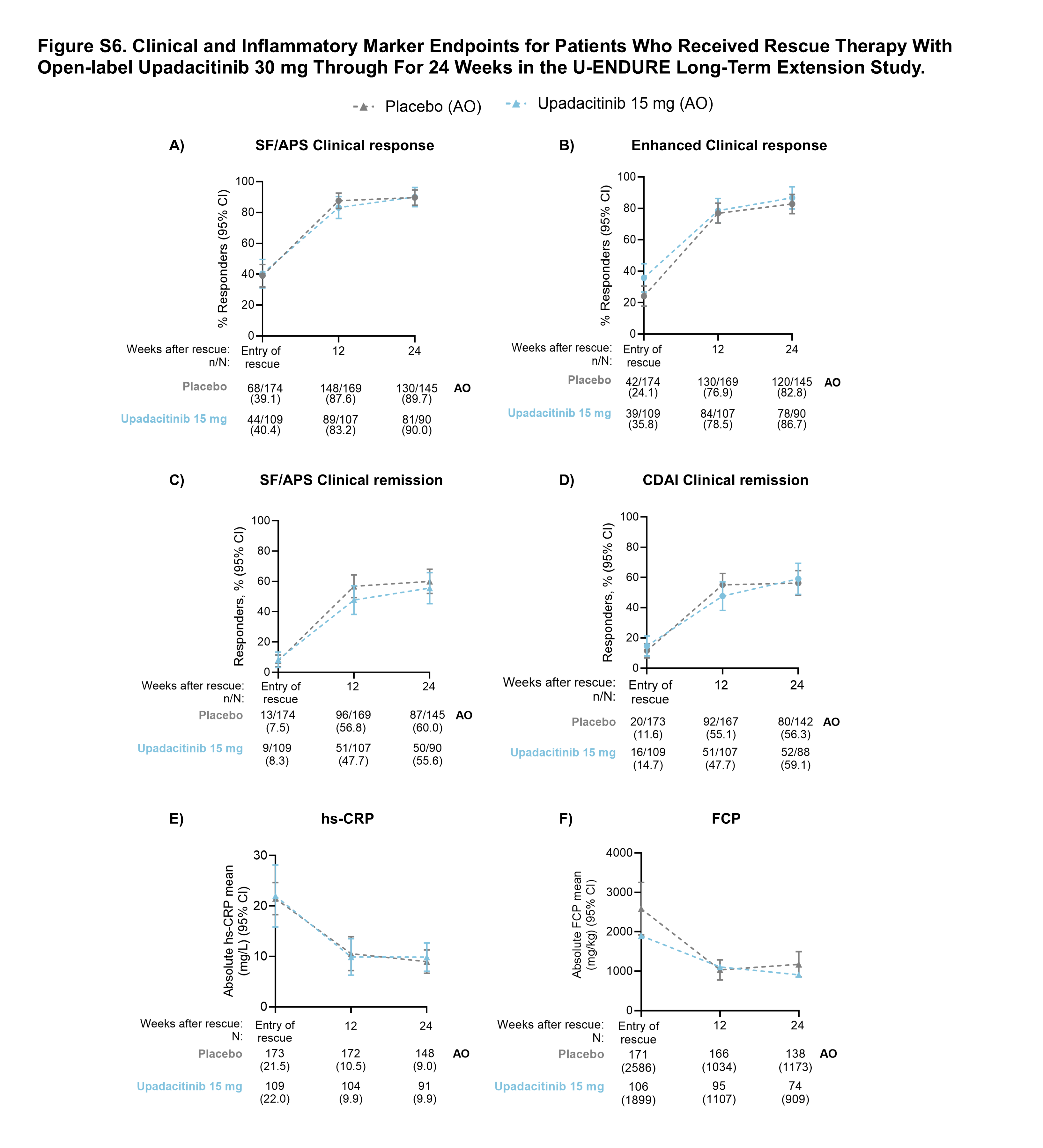

Supplement: jjaf138_Supplementary_Data [file jjaf138_supplementary_data.zip › Figure S6.tif]

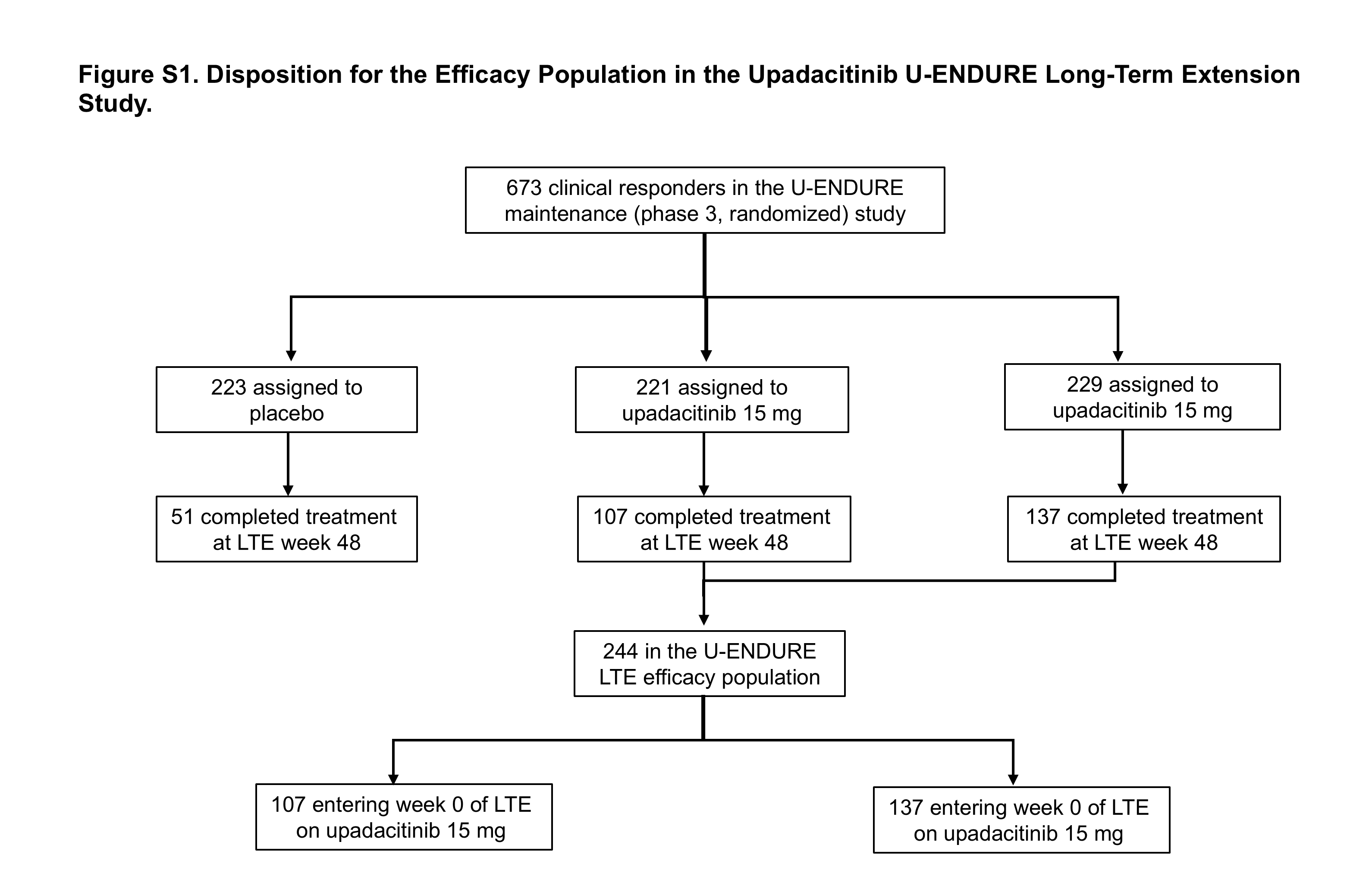

Supplement: jjaf138_Supplementary_Data [file jjaf138_supplementary_data.zip › Figure S1.tif]

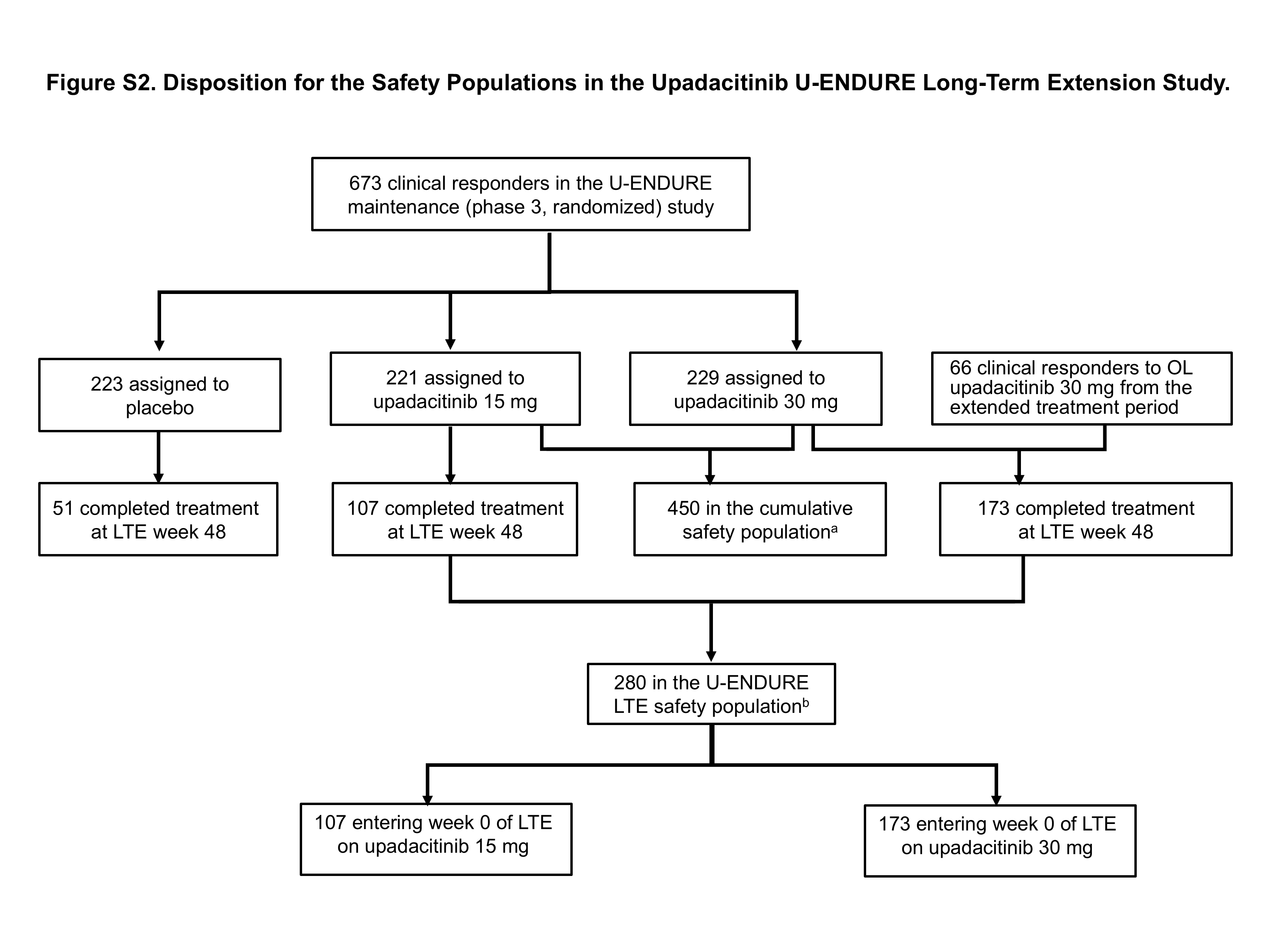

Supplement: jjaf138_Supplementary_Data [file jjaf138_supplementary_data.zip › Figure S2.tif]
